# Analysis of the relationship between short tandem repeats and lactation performance of Xinjiang Holstein cows

**DOI:** 10.1007/s11250-023-03651-y

**Published:** 2023-06-15

**Authors:** Yongqing Li, Li Liu, Abula Zunongjiang, Lijun Cao, Yikai Fan, Bo Hu, Shujun Zhang

**Affiliations:** 1 Key Laboratory of Agricultural Animal Genetics, Breeding and Reproduction (Huazhong Agricultural University), Ministry of Education, Wuhan, 430070 People’s Republic of China; 2 Institute of Animal Husbandry Quality Standard, Xinjiang Academy of Animal Science, Urumqi, Xinjiang, 830063 People’s Republic of China

**Keywords:** Xinjiang, Holstein cow, STR, Lactation performance, Microsatellite loci

## Abstract

Microsatellite markers, also known as short tandem repeats (STRs), are important for marker-assisted selection to detect genetic polymorphism, and they are uniformly distributed in eukaryotic genomes. To analyze the relationship between microsatellite loci and lactation traits of Holstein cows in Xinjiang, 175 lactating cows with similar birth dates, the same parity, and similar calving dates were selected, and 10 STR loci closely linked to quantitative trait loci were used to analyze the correlation between each STR locus and four lactation traits (daily milk yield, milk fat percentage, milk protein percentage, and lactose percentage). All loci showed different degrees of genetic polymorphism. The average values of observed alleles, effective alleles, expected heterozygosity, observed heterozygosity, and polymorphic information content of the 10 STR loci were 10, 3.11, 0.62, 0.64, and 0.58, respectively. Chi-square and G-square tests showed that all populations of loci were in accordance with the Hardy–Weinberg equilibrium. Analysis of the correlation between STR locus genotype and lactation performance in the whole lactation period showed three loci (namely, BM143, BM415, and BP7) with no significant correlation with all lactation traits, two loci (BM302 and UWCA9) related to milk yield, three loci (BM103, BM302, and BM6425) related to milk fat percentage, two loci (BM302 and BM6425) related to milk protein percentage, and three loci (BM1443, BM302, and BMS1943) related to lactose percentage. The microsatellite loci selected in this study showed rich polymorphism in the experimental dairy cow population and were related to the lactation traits, which can be used for the evaluation of genetic resources and early breeding and improvement of Holstein dairy cows in Xinjiang.

## Introduction

Genetic polymorphism, as the basis of animal evolution and development, is an important part of biodiversity and genetic improvement (Williams et al. 1990). Microsatellite markers, also known as short tandem repeats (STRs) or simple sequence repeats (SSRs), are uniformly distributed in the genome of eukaryotes and composed of 1–6 nucleotide tandem repeats, including single type, compound type, and interval type; microsatellite markers are important marker-assisted selection methods, which consists of 1~6 nucleotide tandem repeats, including haplotypes, compound types, and interval types (Takezaki et al. 1996). Microsatellite loci have broad applications in pedigree tracing and gene improvement because of their co-dominant inheritance, rich polymorphism, conservative flanking sequences, and easy design of universal primers. Comprehensive studies performed worldwide have found that quantitative trait loci for milk yield, milk fat, and milk protein located using microsatellite markers in dairy cows are distributed on chromosomes 3, 6, 7, 8, 9, 10, 11, 14, 17, 18, 20, 21, 23, 25, 26, 27, and 28. Yin Bin et al. (2016) selected eight microsatellite loci from the database of the International Society of Animal Genetics to analyze the relationship between their corresponding genotypes and production traits and obtained a molecular basis for early breeding of Holstein cows. In 2018, Polish scientist Dux discovered a microsatellite locus in the intron 23 region of insulin-like growth factor receptor two and significantly associated different genotypes of this locus with high milk yield, high milk fat percentage, and high milk protein level (Dux M et al. 2018). Recently, many studies have analyzed STRs and SSRs in plants and microorganisms, for example, microsatellite loci related to drought tolerance traits in potato (Schumacher et al. 2021), apotheciate *Usnea florida* (Degtjarenko et al. 2020), and resistance to scab in European triticale (Ollier et al. 2020). However, relatively few studies have analyzed STRs and SSRs in animals. In 2022, Griciuvien et al. (2022) used STRs to analyze genetic structure changes in the wild boar (*Sus scrofa*) in Lithuania, following an outbreak of African swine fever. Microsatellite technology has been used to analyze the genetic diversity and genetic bottleneck of buffalo (Ali et al. 2021); genetic identification of Zavot cattle (Boğa et al. 2022); identification of phenotype and genetic diversity of high-altitude yaks in Pakistan (Hameed et al. 2022); relationship between genetic diversity and phylogeny of cattle in Senegal (Sambe et al. 2022) and Siberian black-skinned cattle (Aitnazarov et al. 2021); genetic diversity of cattle in Kerala, India; and relationship between STR genetic diversity and quantitative trait variations of bull semen (Gororo et al. 2021). There has been limited research on the correlation between microsatellite loci and milk production traits of Holstein cows, and there are no reports on microsatellite locus analysis of Holstein cows in Xinjiang. In addition, the correlation analysis results between microsatellite loci and lactation performance obtained by previous researchers were compared, and the correlations between some established loci and traits were found to be inconsistent. To identify and confirm the correlation between microsatellite loci and lactation performance of Holstein cows in Xinjiang, in this study, 10 STR markers and milk production traits (milk yield, milk fat percentage, milk protein percentage, and lactose percentage) of Holstein cows were analyzed in a complete lactation period in Xinjiang. Our findings can be used for the protection and utilization of high-quality genetic resources of Holstein cows in Xinjiang and culling of cows with relatively weak lactation performance.

## Materials and methods

### Animal population

In total, 175 Holstein cows which were born in 2016, first birth, and calving in 2018 stationed at the a large-scale dairy farm in northern Xinjiang were included in the current investigation.

### Collection of phenotypic traits

The FOSS milk composition analyzer (Fossomatic 5000basic 75710, Foss, Denmark) was used to measure four lactation-related traits: milk yield (kg), milk fat percentage, milk protein percentage, and lactose percentage. The traits were measured once a month for 10 consecutive months.

### Blood collection and DNA extraction

Venous blood (3–4 ml) was collected from the base of tail of the cattle in EDTA tubes, shaken well, transferred to 5-ml freezing tubes, and placed in a liquid nitrogen tank for storage. The whole-blood genome was extracted using a DNA extraction kit (Tiangen, DP304-02), and the DNA quality was detected with 0.75% agarose gel electrophoresis; DNA concentration and purity were detected using a spectrophotometer, and the DNA samples were stored at −20 °C.

### Selection and amplification of microsatellite loci

According to the recommendation of the Food and Agriculture Organization of the United Nations (https://www.fao.org/3/i1102t/i1102t.pdf) and the INTERNE database of BOVMAP (http://LOCUS.INRA.FR/CGI-BIN/BOVMAP), 10 microsatellite loci closely adjacent to quantitative trait loci were selected as candidate loci; the information is listed in supplementary file. The primers were synthesized by Shanghai Shenggong Bioengineering Co., Ltd. The amplification volume was 25 μl: 2.5 μl of 10× Taq buffer (with MgCl_2_), 0.5 μl of 10 μm DNTP (mix), 0.5 μl of 10 μmol/l forward and reverse primers, 0.2 μl of 5 U/μl Taq enzyme, and ddH_2_O (up to 25 μl). The optimized thermocycling conditions were as follows: initial denaturation at 95 °C for 5 min; 10 cycles of denaturation at 94 °C for 30 s, annealing at 60 °C for 30 s, and extension at 72 °C for 30 s; 30 cycles of denaturation at 94 °C for 30 s, annealing at 55 °C for 30 s, and extension at 72 °C for 30 s; and preservation at 4 °C.

### STR detection

A mixture of 990 μl Hi-Di Formamide and 10 μl Liz 500 was added to a 96-well reaction plate and centrifuged for 15 s at 10 μl and 1200 rpm per well; then, 1 μl of the amplified sample was added and centrifuged for 15 s at 1200 rpm after shaking. Denaturation was performed at 98 °C for 5 min, and the 96-well plate was immediately placed in an ice-water mixture and cooled rapidly. Capillary electrophoresis was performed with an ABI sequencer (3730XL). The GeneMapper software was used to analyze the STR data. The alleles were numbered A–V, according to fragment lengths.

### Genetic diversity analysis

Multi-population descriptive statistics were conducted using PopGen32 software. The statistical genetic parameters included observed allele number (Na), effective allele number (Ne), Shannon index (*I*), expected heterozygosity (*H*_e_), observed heterozygosity (*H*_o_), and polymorphic information content (PIC).

### Variance analysis between STR variation and lactation performance

SPSS 27.0 was used for multivariate variance analysis of the general linear model, and variance analysis of different genotypes and milk production traits was conducted. The general linear model was as follows:

$${Y}_{ij}=U+{G}_i+{e}_{ij}$$

where *Y*_ij_ is the *j*th measured value of milk production traits of the *i*th genotype; *U* is the population average; *G*_i_ is the fixed effect of the *i*th genotype; and *E*_ij_ is the random residual effect.

## Results

### Detection of amplified products at different STR loci and results of capillary electrophoresis

The PCR amplification products of 10 microsatellite loci were detected using 1% agarose gel electrophoresis. The target bands were clear and bright, and the fragment sizes met our expectations. The PCR products were detected using capillary electrophoresis with the ABI3730 sequencer. The results are listed in supplementary file.

### Allele fragment length and genotype frequency of different STR loci

The sequencing results of STR loci were sorted and screened, and 108 alleles were observed at 10 STR loci. The highest number of alleles was detected at BM1443 (22 alleles) and the lowest numbers at BM143, BMS1943, BM302, and BP7 (5, 6, 7, and 7, respectively). Statistical analysis was made on genotypes with four or more individuals in microsatellite loci; the results of analysis are shown in supplementary file.

### Genetic diversity analysis

The Na, Ne, *I*, *H*_e_, *H*_o_, and PIC of each microsatellite locus calculated using PopGen32 software are listed in supplementary file. The average of Na was 10, and Ne was 3.11. The highest *H*_o_ (BM103) and lowest *H*_o_ (BM143) were 0.81 and 0.02, respectively. The highest *H*_e_ (BM103) and lowest *H*_e_ (BM143) were 0.78 and 0.12, respectively. The PIC ranged from 0.11 (BM143) to 0.74 (BM103); 2 loci, namely, BM143 and BM1443, were less than 0.5, which indicated that the polymorphism of these 10 microsatellite loci was relatively rich. The chi-square and G-square test results are shown in supplementary file. The results showed no significant differences between *H*_o_ and *H*_e_ of the 10 loci, except BM143, which is consistent with the Hardy–Weinberg equilibrium.

### Association analysis of different STR loci genotypes with lactation traits

Multivariate analysis of variance of the general linear model was conducted using SPSS 27.0. The results showed that, among the 10 microsatellite loci, seven loci were related to lactation traits, whereas the other three loci, BM143, BM415, and BP7, had no correlation with lactation traits. Histograms of the differential analysis of lactation traits of individuals with different genotypes were created using GraphPad Prism 5 software.

### Microsatellite loci related to milk fat

**Fig. 1:**
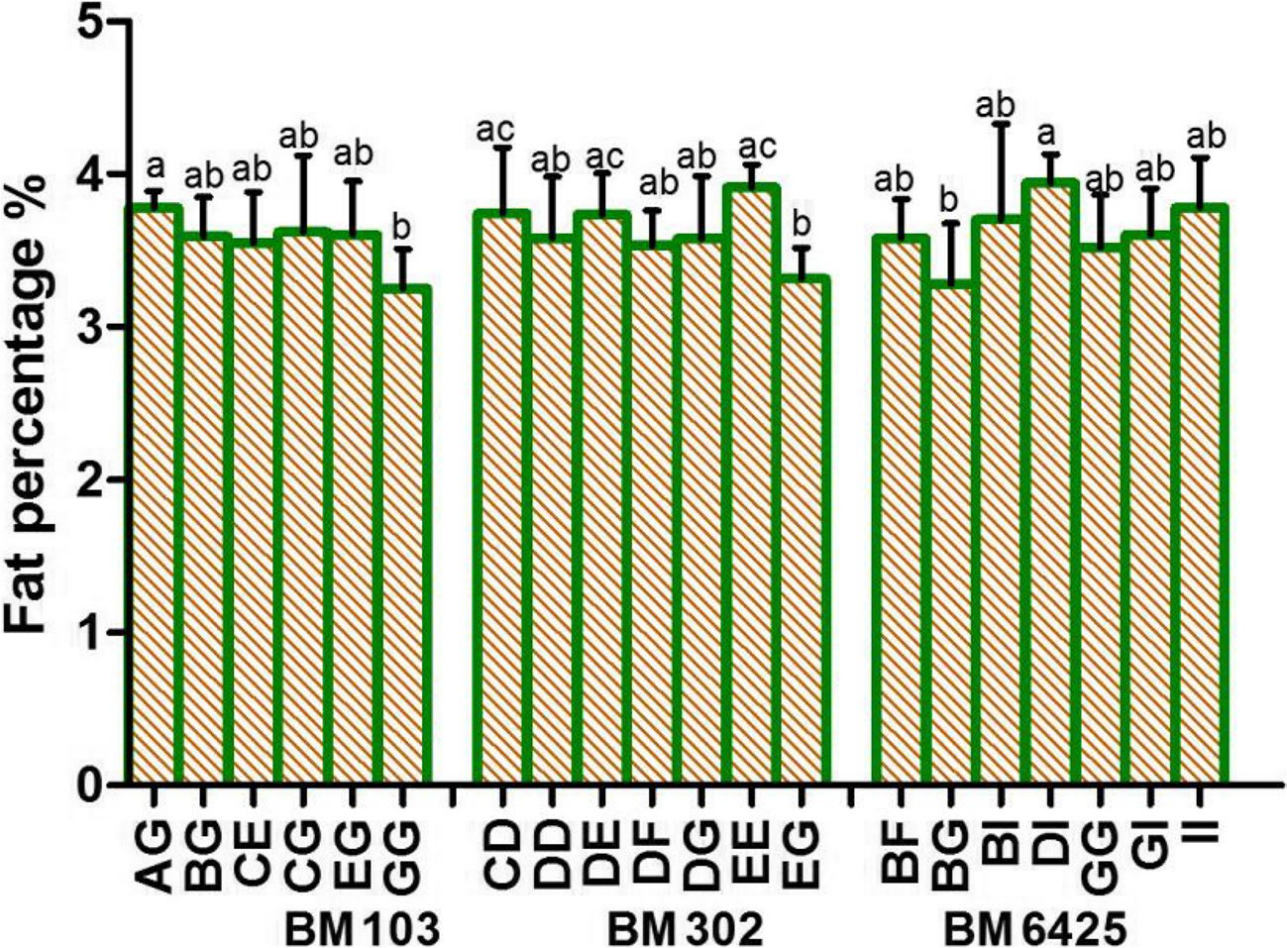
Analysis of differences in milk fat percentage of individuals with different genotypes at the BM103, BM302, and BM6425 loci

Three loci (BM103, BM302, and BM6425) were related to fat percentage (Fig. 1; Table 1). A significant difference was observed between AG genotype and GG genotype at BM103 locus (*P* < 0.05), which indicates that allele A has a positive effect on fat percentage when compared with allele G. At the BM302 locus, significant differences were found between individuals with CD, DE, and EE genotypes and individuals with EG genotypes (*P* < 0.05) for fat percentage, which indicates that G allele has a negative effect on fat percentage. A significant difference in fat percentage was detected between DI genotype individuals (3.94%) and BJ genotype individuals (3.28%; *P* < 0.05) at the BM6425 locus.

**Table 1:** Differences in the lactation traits of different genotypes of the Holstein cows at the microsatellite loci

Marker genotypes	Fat %	Protein %	Lactose %	Milk yield
BM103				
AG (16)	3.78 ± 0.11^a^	-	-	-
BG (32)	3.59 ± 0.26^ab^	-	-	-
CE (12)	3.55 ± 0.33^ab^	-	-	-
CG (28)	3.62 ± 0.5^ab^	-	-	-
EG (32)	3.6 ± 0.35^ab^	-	-	-
GG (24)	3.25 ± 0.26^b^	-	-	-
BM1443				
CI (28)	-	-	5.15 ± 0.1^ab^	-
DI (36)	-	-	5.12 ± 0.12^ab^	-
GJ (16)	-	-	5.23 ± 0.15^a^	-
JQ (28)	-	-	5.05 ± 0.16^b^	-
JT (16)	-	-	5.22 ± 0.09^a^	-
JV (16)	-	-	5.2 ± 0.12^ab^	-
BM203				
BE (32)	-	-	5.15 ± 0.1^ab^	-
BH (39)	-	-	5.12 ± 0.12^ab^	-
BN (17)	-	-	5.23 ± 0.15^a^	-
HN (32)	-	-	5.05 ± 0.16^b^	-
MM (15)	-	-	5.22 ± 0.09^a^	-
NN (17)	-	-	5.2 ± 0.12^ab^	-
BM302				
CD (24)	3.74 ± 0.43^a^	3.2 ± 0.11^ab^	-	31.85 ± 7.63^bc^
DD (30)	3.58 ± 0.4^ab^	3.17 ± 0.16^ab^	-	35.08 ± 3.77^ab^
DE (24)	3.73 ± 0.27^a^	3.27 ± 0.15^a^	-	31.4 ± 4.15^bc^
DF (15)	3.53 ± 0.23^ab^	3.16 ± 0.07^ab^	-	33.48 ± 5.65^abc^
DG (27)	3.58 ± 0.41^ab^	3.22 ± 0.12^ab^	-	33.48 ± 6.33^abc^
EE (12)	3.91 ± 0.15^a^	3.35 ± 0.32^a^	-	27.13 ± 7.28^c^
EG (21)	3.32 ± 0.2^b^	3.09 ± 0.2^b^	-	38.24 ± 4.6^a^
BM6425				
BF (24)	3.58 ± 0.25^ab^	3.15 ± 0.07^c^	-	-
BG (16)	3.28 ± 0.4^b^	3.16 ± 0.2^c^	-	-
BI (20)	3.7 ± 0.63^ab^	3.39 ± 0.23^a^	-	-
DI (16)	3.94 ± 0.19^a^	3.1 ± 0.1^c^	-	-
GG (24)	3.52 ± 0.34^ab^	3.15 ± 0.17^c^	-	-
GI (20)	3.6 ± 0.3^ab^	3.17 ± 0.2^bc^	-	-
II (20)	3.78 ± 0.33^ab^	3.29 ± 0.11^abc^	-	-
BMS1943				
CE (30)	-	-	5.15 ± 0.08^ab^	-
CF (30)	-	-	5.15 ± 0.15^ab^	-
EE (36)	-	-	5.17 ± 0.1^a^	-
EF (48)	-	-	5.16 ± 0.1^a^	-
FF (12)	-	-	5.03 ± 0.18^b^	-
UWCA9				
DD (18)	-	-	-	37.78 ± 4.91^a^
DK (12)	-	-	-	34.73 ± 6.39^ab^
EE (21)	-	-	-	33.13 ± 4.09^ab^
EF (12)	-	-	-	31.53 ± 7.5^ab^
EJ (18)	-	-	-	35.42 ± 4.79^ab^
EK (33)	-	-	-	31.21 ± 5.56^b^
EL (18)	-	-	-	33.27 ± 6.94^ab^

Expressions not listed in the table have no correlation with the trait at this locus. Different lowercase letters in the same column indicate significant differences (*P* < 0.05). The same letter or no letter indicates no significant difference (*P* > 0.05)

### Microsatellite loci related to milk protein

**Fig. 2:**
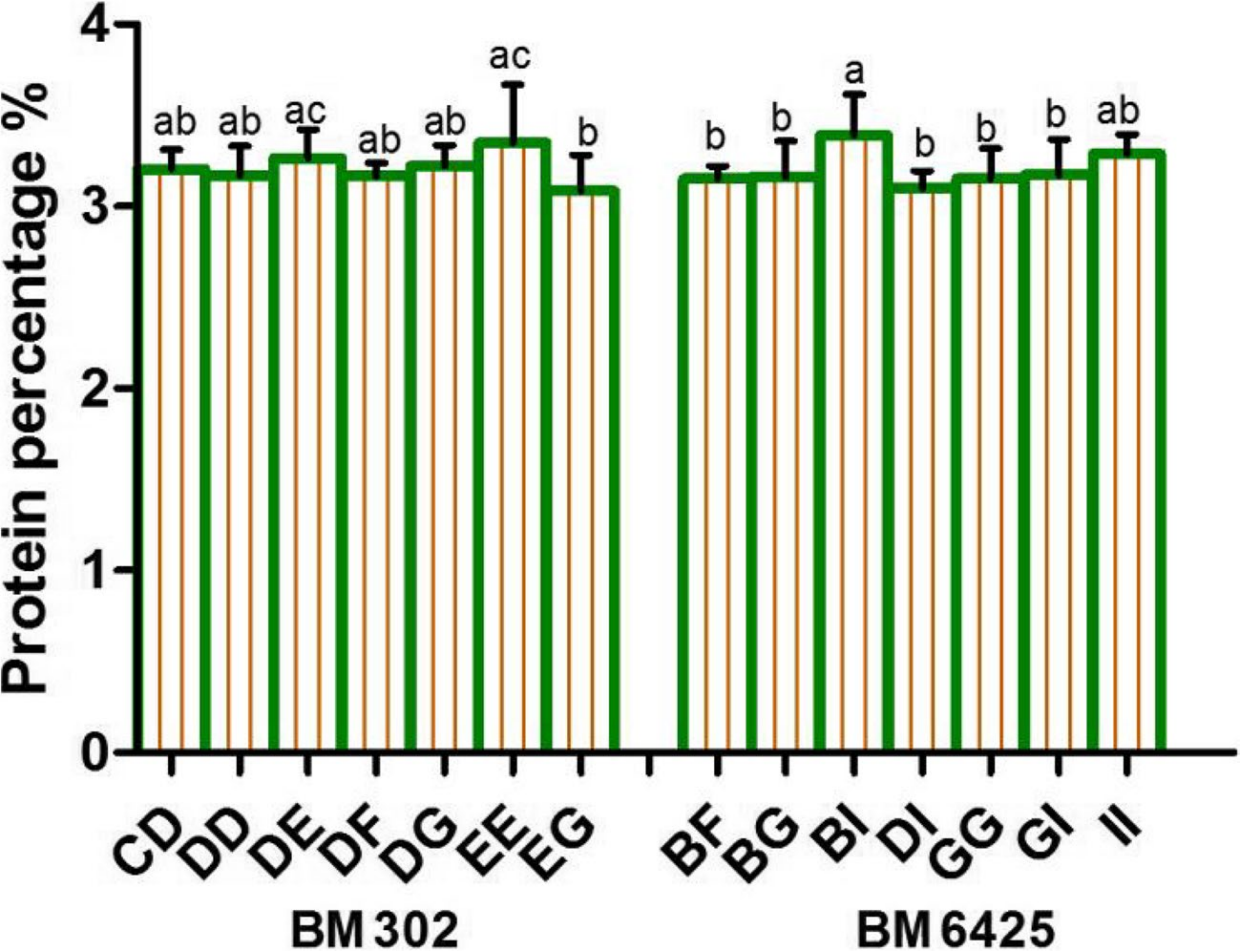
Analysis of protein percentage differences among individuals with different genotypes at the BM302 and BM6425 loci

Two loci (BM302 and BM6425) were related to protein percentage (Fig. 2; Table 1). Significant differences were observed between DE and EE genotype individuals and EG genotype individuals at the BM302 locus (*P* < 0.05) for protein percentage, which indicates that G allele has a negative effect. A significant difference in protein percentage was found between BI genotype individuals and BF, BG, DI, GG, and GI genotype individuals at the BM6425 locus (*P* < 0.05).

### Microsatellite loci related to milk lactose

**Fig. 3:**
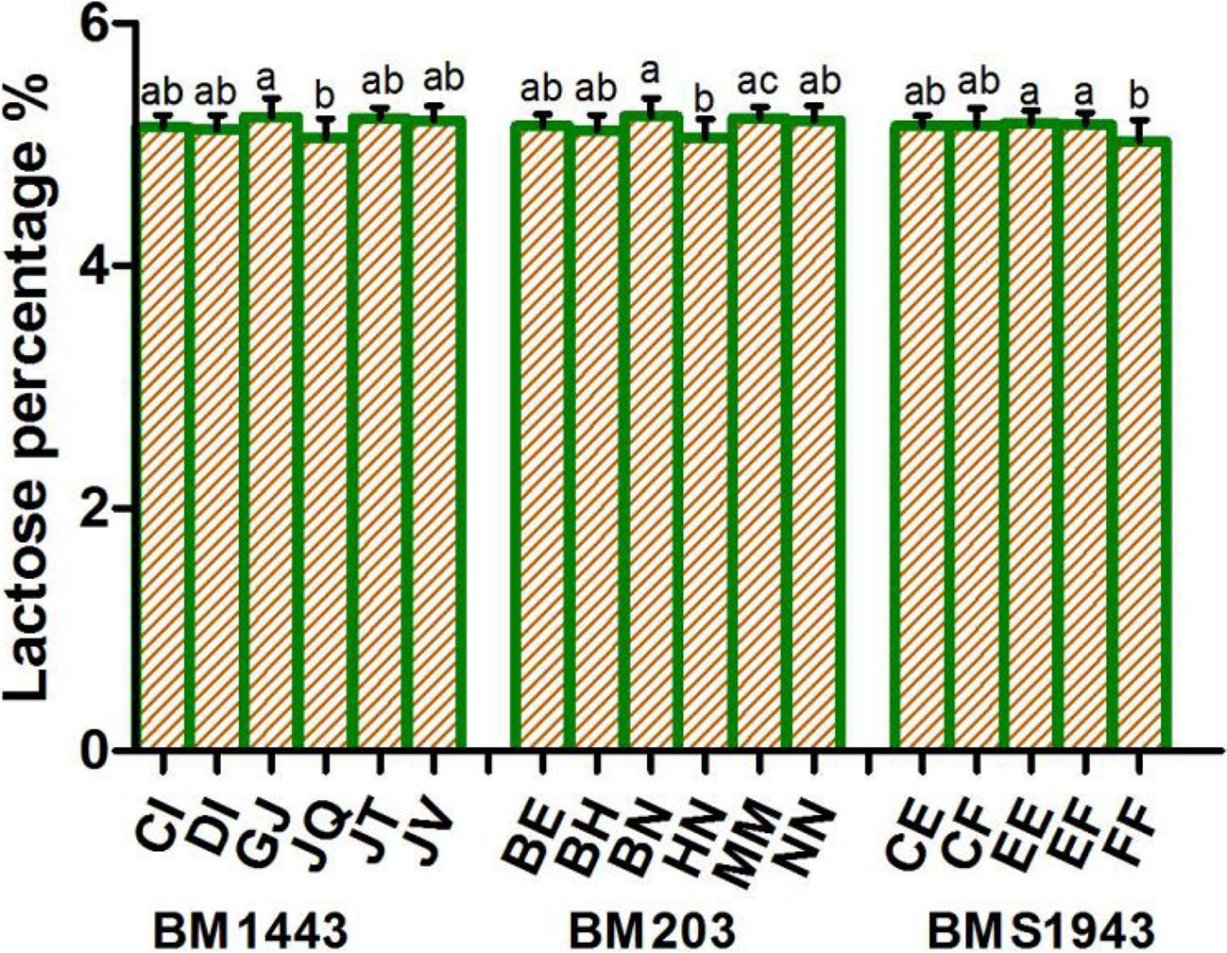
Analysis of lactose percentage differences among individuals with different genotypes at the BM1443, BM203, and BMS1943 loci

Three loci (BM1443, BM302, and BMS1943) were related to lactose percentage (Fig. 3; Table 1). A significant difference was observed between GJ and JQ genotypes at the 1443 locus (*P* < 0.05), which indicates that allele G has a positive effect on lactose percentage when compared with allele Q. At the BM203 locus, a significant difference was found between BN and HN genotypes (*P* < 0.05) and between HN and MM genotypes (*P* < 0.05), which indicates that allele B has a positive effect on lactose percentage when compared with allele H and allele M also has a positive effect. A correlation was found between the BMS1943 locus and lactose percentage. A significant difference in lactose percentage was observed between EE and EF genotype individuals and FF genotype individuals (*P* < 0.05), indicating that allele E has a positive effect and allele F has a negative effect on lactose percentage.

### Microsatellite loci related to milk yield

**Fig. 4:**
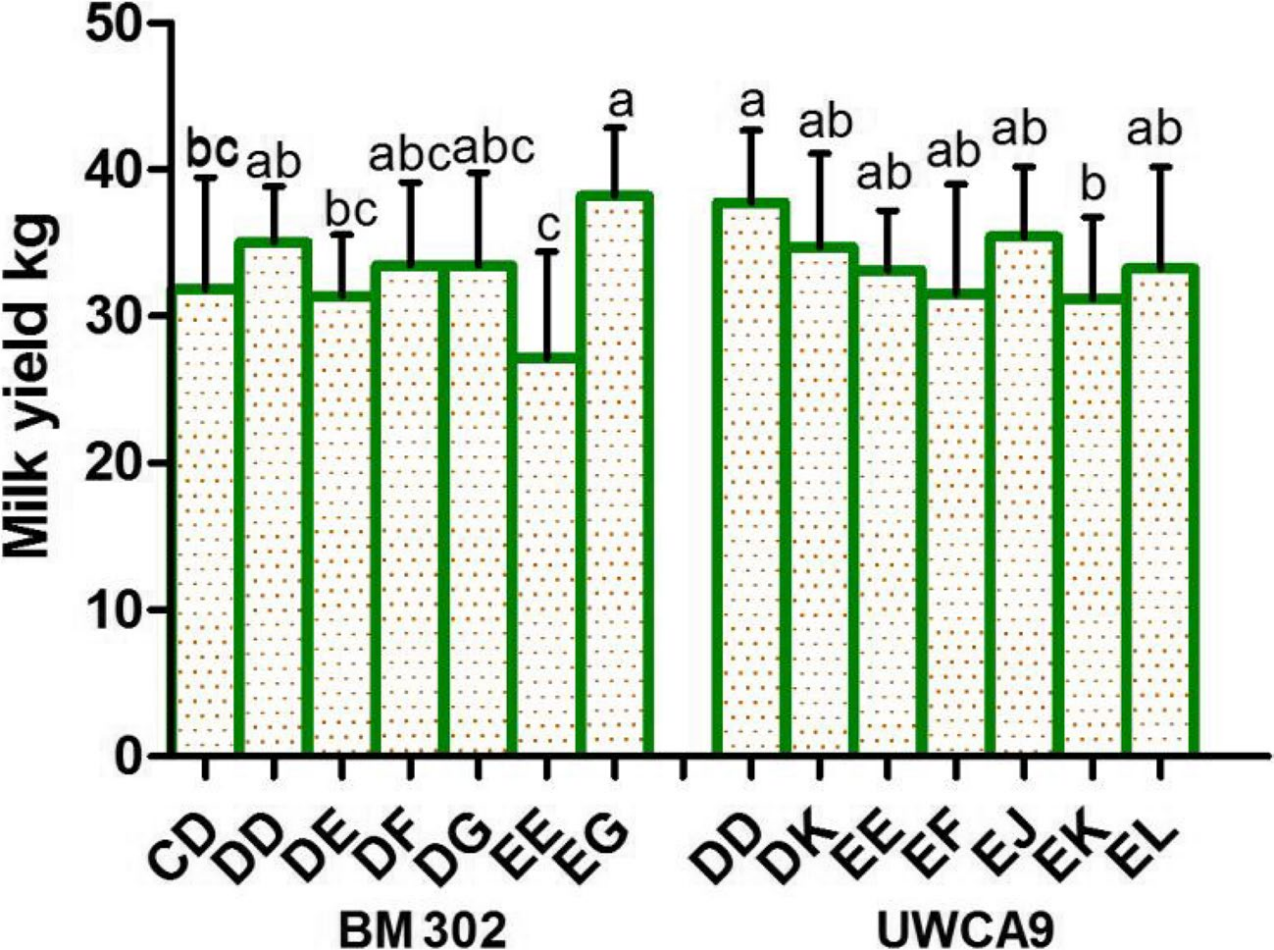
Analysis of milk yield differences among individuals with different genotypes at the BM302 and UWCA9

Two other loci (namely, BM302 and UWCA9) were found to be related to milk yield (Fig. 4; Table 1). Significant differences were detected between EG genotype individuals and CD, DE, and EE genotype individuals (*P* < 0.05) and between DD and EE genotype individuals (*P* < 0.05) at the BM302 for milk yield. A significant difference in milk yield was observed between DD genotype individuals and EK genotype individuals at the UWCA9 locus (*P* < 0.05), which indicates that allele D has a positive effect on milk yield.

## Discussion

### Genetic parameter analysis

*H*_o_ and *H*_e_ are the best indicators to measure the degree of genetic variation in a population (Wang et al. 2007). In Indian water buffaloes, Vani et al. (2022) found that *H*_o_ of the BM415 locus was 0.097, which is far lower than 0.76 in this study; this indicated that the polymorphism of dairy cows was abundant at this locus. In this study, the average H_o_ and H_e_ values were 0.64 and 0.62, respectively; the values are close to each other, indicating that the genotype distribution of the experimental population is close to equilibrium. In this study, the maximum and minimum Na values were 22 (BM1443 locus) and 5 (BM143), respectively, and the maximum and minimum Ne values were 4.41 (BM103) and 1.13 (BM143), respectively. The difference between Ne and Na was large, indicating that the distribution of alleles in some loci is uneven. In this study, 3 effective alleles were found at the UWCA9 locus, which is lower than the 5 reported by Vani et al. (2022). When this study was compared with that by Vani et al. (2022), differences in Na were observed at the BM1443 locus (22 and 4, respectively). The differences in Na may be caused by the differences in the number of samples and change in components.

PIC refers to the value of a marker used to detect polymorphism in a population. The value depends on the number of detected alleles and their frequency distribution (Nei, 1987), and it is calculated using PIC_CALC 0.6 software (Sambe, 2022). The results of this study showed that the 10 microsatellite loci have low to moderate polymorphism in Holstein dairy cattle, and the PIC ranged from 0.11 (BM143) to 0.74 (BM103). In the genetic analysis of a population, genetic markers with PIC value more than 0.5 are usually regarded as more informative (Botstein et al. 1980). The average PIC value of all loci in this study was 0.58, indicating that, overall, the polymorphism was abundant.

### Correlation analysis

Vani et al. (2022) used 21 microsatellite loci of dairy cows to study the relationship with the lactation traits of buffalo. Among them, three microsatellite loci (BM1443, BM415, and BM143) were the same as those used in this study, but the results are inconsistent. Vani et al. (2022) found no significant correlation between the BM1443 locus and lactation performance of water buffalo (*P* > 0.05). In this study, the BM1443 locus was significantly correlated with lactose percentage (*P* < 0.05). The results for BM415 and BM143 loci were consistent with those of this study, with no significant correlation. Van et al. (2000) thought that BM415 and BP7 were significantly correlated with protein percentage, but these two loci were not correlated with protein percentage in this study. The results of correlation between the BM302 locus and lactation traits were consistent with those of Zhao et al. (2010), and this locus may be significantly correlated with milk yield, fat percentage, and protein percentage (*P* < 0.05). When the effects of UWCA9 on lactation traits were analyzed, the results of this study were inconsistent with those of Guo et al. (2007) but consistent with those of Vilkki et al. (1997). Guo et al. (2007) reported that UWCA9 has an influence on fat percentage and protein percentage, but we and Vilkki et al. (1997) found that UWCA9 has an influence on only milk yield and no correlation with other traits. In this study, the effects of BM103 and BM302 on fat percentage were consistent with the results of Ashwell et al. (1997). In addition, we found a new locus (BM302) significantly related to protein percentage and three loci (BM1443, BM302, and BMS1943) significantly related to lactose percentage.

## Conclusions

The correlation analysis showed three loci (namely, BM143, BM415 and BP7) with no significant correlation with all lactation traits. Two other loci (namely, BM302 and UWCA9) were found to be related to milk yield, three loci (BM103, BM302, and BM6425) were related to fat percentage, two loci (BM302 and BM6425) were related to protein percentage, and three loci (BM1443, BM302, and BMS1943) were related to lactose percentage. However, the number of experimental animals was an important limiting factor. In the future, it will be necessary to increase the number of experimental cattle, sample size, and microsatellite markers and constantly track the correlation between microsatellite loci and milk production performance of Holstein cows, to obtain consistent microsatellite markers for screening excellent milk production traits of Holstein cows.

## Data Availability

All data generated or analysed during this study are included in this published article and its supplementary information files.

## Data Availability

All data generated or analysed during this study are included in this published article and its supplementary information files.
